# Cannabidiol Determination on Peripheral Capillary Blood Using a Microsampling Method and Ultra-High-Performance Liquid Chromatography Tandem Mass Spectrometry with On-Line Sample Preparation

**DOI:** 10.3390/molecules25163608

**Published:** 2020-08-08

**Authors:** Federica Pigliasco, Sebastiano Barco, Sara Dubois, Francesca Marchese, Pasquale Striano, Tommaso Lomonaco, Francesca Mattioli, Gino Tripodi, Giuliana Cangemi

**Affiliations:** 1 Chromatography and Mass Spectrometry Section, Central Laboratory of Analyses, IRCCS Istituto Giannina Gaslini, 16147 Genoa, Italy; f.pigliasco@gmail.com (F.P.); SebastianoBarco@gaslini.org (S.B.); ginotripodi@gaslini.org (G.T.); GiulianaCangemi@gaslini.org (G.C.); 2 Department of Neurosciences, Rehabilitation, Ophthalmology, Genetics, Maternal and Child Health, University of Genoa, 16132 Genoa, Italy; sara.dubois@libero.it; 3 Pediatric Neurology and Muscular Diseases Unit, IRCCS Istituto Giannina Gaslini, 16147 Genoa, Italy; FrancescaMarchese@gaslini.org; 4 Department of Chemistry and Industrial Chemistry, University of Pisa, 56124 Pisa, Italy; tommaso.lomonaco@unipi.it; 5 Department of Internal Medicine, Pharmacology and Toxicology Unit, University of Genoa, 16132 Genoa, Italy; fmattiol@unige.it

**Keywords:** cannabidiol, microsampling, therapeutic drug monitoring, finger prick

## Abstract

The aim of this work is to evaluate volumetric absorptive microsampling (VAMS) from capillary blood as an alternative strategy for therapeutic drug monitoring (TDM) in patients treated with the newly available GW-purified form of cannabidiol (Epidiolex^®^). A fast ultra-high-performance liquid chromatography-tandem mass spectrometry (UHPLC-MS/MS) coupled to an online sample preparation system analysis was carried out on a Thermo Scientific Ultimate 3000 LC system coupled to a TSQ Quantiva triple quadrupole for the quantification of cannabidiol (CBD) and, in addition, delta-9-tetrahydrocannabinol (Δ9-THC). After validation using European Medicine Agency (EMA) guidelines the method was applied to samples obtained by finger prick of five pediatric patients treated with Epidiolex^®^ and the results were compared to those obtained from venous blood and plasma. The method is linear in the range of 1–800 µg/L for both CBD and THC with intra- and inter-day precisions ranging from 5% to 14% and accuracies from −13% to +14% starting from 30 µL of sample. Stability in VAMS is ensured for up to 4 weeks at 25 °C thus allowing simple delivery. There was no difference (*p* = 0.69) between concentrations of CBD measured from VAMS sampled from capillary or venous blood (range: 52.19–330.14 or 72.15–383.45 µg/L) and those obtained from plasma (range: 64.3–374.09 µg/L) The VAMS-LC-MS/MS method represents a valid alternative strategy for therapeutic drug monitoring of patients treated with Epidiolex^®^.

## 1. Introduction

Therapeutic drug monitoring (TDM) is helpful in the management of patients with epilepsy for old and new anti-seizure medications (ASMs), often in response to single patient-specific pharmacokinetic or pharmacodynamic issues [1,2,3]. TDM in epilepsy patients is commonly performed on plasma or serum; however, the use of dried plasma spots (DPSs) may facilitate sample shipment to reference laboratories [4,5].

The growing interest in cannabis-based therapies has increased in recent years for various clinical applications among which treatment of drug-resistant epilepsy is one of the most relevant [6,7,8]. Cannabis is a complex matrix made approximately by 500 compounds among which at least 90 cannabinoids [9]. Δ9-tetrahydrocannabinol (THC) is the main compound responsible for the psychotropic activity of cannabis while cannabidiol (CBD) is the main non-psychoactive constituent which has shown a wide range of therapeutically promising pharmacological effects [10]. 

In recent years, a broad spectrum of products containing CBD has emerged on the market although their effects are largely dependent on the purity, the preparation, and the concentration of CBD and other components [9,10]. A plant-derived pharmaceutical formulation of purified CBD oral solution (Epidiolex^®^) was approved in June 2018 by the US Food and Drug Administration (FDA) as treatment and in July 2019 by the European Medicines Agency as adjunctive therapy in conjunction with clobazam (CLB) for seizures associated with Dravet Syndrome (DS) or Lennox-Gastaut syndrome (LGS) for patients aged 2 years and older [11].

The pharmacokinetics (PK) of CBD shows extensive variability depending on the route of administration (e.g., intravenous, oral, sublingual, oromucosal spray, inhalation, and transdermal), type of product administered, concomitant intake of food, drug-drug interactions, and other factors [9,10,11]. A relationship between administered dose and blood levels has been demonstrated in some studies [12,13,14] laying the bases for TDM to optimize CBD based therapy in epilepsy patients [15]. However, a major challenge in practicing TDM in patients on CBD is the discomfort caused by repeated venipunctures, especially in children. The availability of patient-friendly techniques for the measurement of CBD from capillary blood obtained by finger prick would facilitate its use for TDM, opening a new way for personalized precision medicine. Compared to blood, the use of dried blood spot or other microsampling techniques has several advantages: (i) fast, easy and minimally invasive sample collection, (ii) low sample volume, typically in the range 10–50 μL, (iii) minimal sample manipulation and shipping requirements, (iv) safe handling of samples with minimum risks of transmission of infectious diseases [16].

Herein, we describe a new analytical approach based on a microsampling method and liquid chromatography coupled to tandem mass spectrometry (LC-MS/MS) for the determination of CBD and THC in blood samples. We adopted a volumetric absorptive microsampling (VAMS) device, which allows the simple collection of an accurate volume of blood [17]. Compared to the classical non-volumetric-based dried blood spots (DBS) analysis, the performances of this device are not influenced by the hematocrit (HCT) values and by the erythrocyte volume fraction [17,18,19,20,21]. VAMS approach has been yet adopted for several quantitative methods for several molecules in different fields [19,22,23].

The method has been validated using EMA guidelines [24] and the analytical performances of the new VAMS-LC-MS/MS approach were preliminary tested by monitoring applied CBD and THC in blood samples derived from pediatric patients under therapy with the FDA-approved prescription of CBD produced by GW pharma (Epidiolex^®^). The results were also compared to those obtained from the plasma, which is the gold standard matrix due to the high protein binding with human serum albumin (HSA) [25].

## 2. Results

### 2.1. Method Development

Different screening assays were carried out to optimize the extraction conditions from VAMS. Two different organic solvents (methanol and acetonitrile) were then used with or without the addition of a sonication step. The extraction procedure that allowed to obtain the best results in terms of extraction recovery (ER) is described in the materials and methods sections. The re-hydration step, differently from the sonication step, did not significantly improved the ER probably due to the hydrophobic interactions between the polymer material and the analytes.

The LC-MS/MS method that we have previously published for plasma [26] was slightly modified by adding an on-line sample cleanup step using a TurboFlowTM column. Different flow rates (from 1 to 1.5 mL/min) and different percentages of phase A (from 50% to 100%) were tested to optimize the on-line sample cleanup step: the best-operating conditions were obtained at a flow rate of 1.5 mL at 100% phase B. The high linear velocities created by mobile phase flowing through the column particles of the TurboFlowTM column, are at the basis of the efficient purification from proteins and other interferents, that may enhance the signal-to-noise ratio.

### 2.2. Method Validation

**Figure 1:**
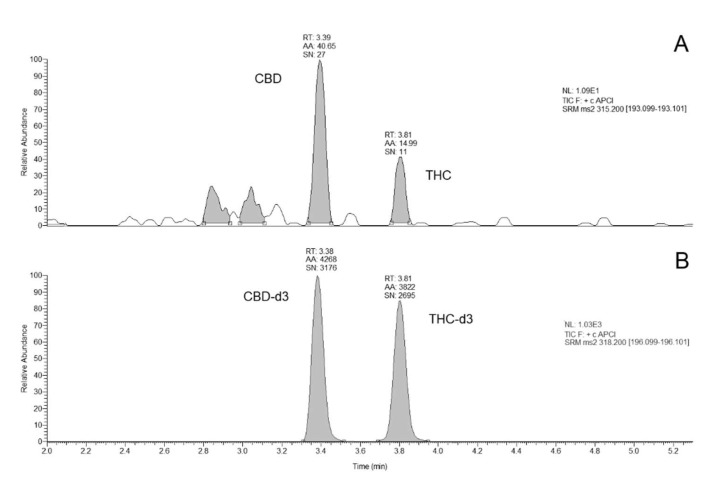
Chromatograms obtained: a calibrator at the LLOQ (panel **A**); deuterated internal standards (panel **B**). RT, retention time. AA, automatic area, SN, signal to noise ratio. NL, normalized level.

The method was validated following EMA guidelines [24] and showed excellent analytical figures of merit. In details, no interfering peaks at the specified LC-MS/MS conditions were observed. Carry-over was negligible. For both THC and CBD, the lower limit of quantification (LLOQ) resulted 1.0 µg/L. Figure 1 shows representative chromatograms obtained. A linear relationship between the analytes peak area and the corresponding concentration was achieved in the entire concentration range (with r^2^ = 0.99) and the back-calculated concentration values for both analytes were not significantly different to the theoretical value (±15%). Results of the intra- and inter-assay precision and accuracy and recoveries complied all the acceptable ranges suggested by the EMA guidelines (Table 1). 

The sample dilution (2 and 5-fold with methanol) did not significantly impact the analytes determination since the estimated concentration agreed within ±15 % of the theoretical value. Matrix effect tests gave results inside the acceptable ranges (in the range 5–11% for CBD and 7–10% for THC). Extraction recovery tests gave 25% for CBD and 24% for THC, in both cases CV% are less than 15%. Recoveries obtained are quite low probably because, to avoid an excessive dilution, we have chosen a low volume of solvent compared to VAMS surface.

Short-term and long-term stability tests (Table 2) demonstrated that both THC and CBD were stable in VAMS at all the temperatures analyzed after 1 week and up to 4 weeks.

### 2.3. Analyses of Clinical Samples

Table 3 summarizes the patients’ characteristics, dose administered and results obtained from venous VAMS, capillary VAMS and plasma samples. The number of patients was low, but it can be considered acceptable in this proof of concept work as it refers to a drug used for compassionate use in a very rare disease and it was not possible to collect more samples.

All the samples were tested in two different analytical runs to evaluate the incurred sample reanalysis precision as suggested by EMA guidelines. The results (inter-day RSD = 12%) confirmed acceptable reproducibility.

**Figure 2:**
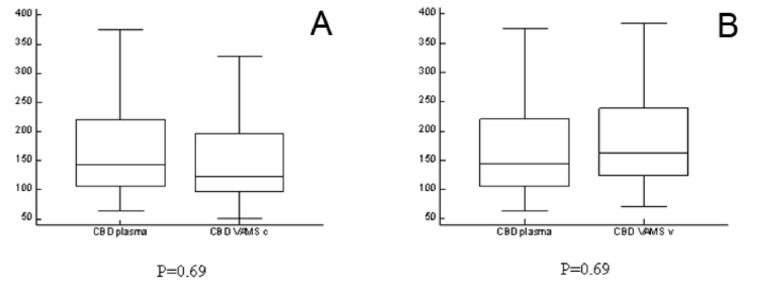
Mann Whitney tests of VAMS from capillary versus venous blood (Panel **A**) and VAMS from capillary blood versus plasma (Panel **B**).

Results obtained from capillary blood were not statistically different from those obtained from venous blood (P = 0.69) or plasma (P = 0.69). Figure 2 shows the results of the Mann Whitney tests.

## 3. Discussion

Several analytical methods are available in the literature for the measurement of THC and CBD from various biological matrices such as urine, saliva or hair [26,27,28,29,30].

Few of them [28,29,31,32,33] have been validated for their suitability for TDM of medical cannabis on plasma samples. The disadvantage of TDM based on plasma samples is represented by the discomfort caused to patients to obtain venous samples by using sampling procedures that cannot be applied at all to pediatrics patients and/or newborns, limiting the widespread of TDM in such population. To overcome these issue, new microsampling devices are described in literature [34,35,36]. These systems allow to collect a known volume of blood by means of microfluidic system, nullifying the HCT and drop volume effects on the analytes determination [17,19,20,21]. Herein, we describe a new microsampling method for the simultaneous determination of THC and CBD using VAMS which has been demonstrated useful for the quantitative measurement of several drugs from venous and capillary blood including first-generation and third-generation antiseizure drugs [23,37], antibiotics [22] and immunosuppressants [35,36,37].

As example, Mercolini et al. [38] demonstrated the applicability of VAMS for the measurement of natural and synthetic cannabinoids including THC and its 2 major metabolites (THC-OH and THC-COOH) for forensic and sports drug testing purposes.

In this study, we focused on VAMS for the measurement of CBD for the TDM of medical cannabis. VAMS was coupled to an LC-MS/MS method with an online sample clean up procedure to enable a specific, accurate, and reproducible quantification of THC and CBD starting from a 30 µL drop of blood.

The new VAMS-LC-MS/MS method has been validated following international guidelines to verify its performance and it assures rapid and efficient quantification of CBD and THC displaying high specificity, accuracy (ranging from −1 to +14%), reproducibility (ranging from +5 to +14%) and linearity covering a wide range of concentrations (1–800 µg/L).

Recently, another paper describing an LC-MS/MS method for quantification of THC, CBD and cannabinol (CBN) from VAMS for application to a pharmacokinetic study has been published [36]. Differently from our, the method from Moorthy et. al. was based on time consuming off-line solid phase extraction. Here we propose the use of an online sample clean-up procedure which to simplify and automatize the sample preparation protocol. Another advantage of the proposed approach is the possibility to reuse the on-line sample cleanup TurboFlow^TM^ column, increasing the laboratory productivity and reducing in turn the cost of each analysis. At the moment of compiling this paper we have performed up to 500 injections without any drop in column performance.

Moreover, the high stability of samples at room temperature of the analytes, may facilitate the storage of samples, leading to minimize the risks of infections and to reduce shipment costs.

Differently from the findings from Moorthy et. al., in our cohort, the concentration of CBD obtained from capillary blood sampled by VAMS did not differ from those obtained from venous blood and plasma [37,39,40]. This may be due to the different approach used as we employed real samples obtained from patients to which CBD was administered in a clinical setting, while the comparison of Moorthy et. al. was based on QC samples obtained “in vitro”. In addition, Moorthy et. al. showed a PK profile of a patient under treatment with CBD without giving any information of the CBD formulation and sampling procedure from patients. In our paper we provide, for the first time, data obtained on real patient’ samples under treatment with Epidiolex^®^ obtained with VAMS on capillary blood.

Our findings support the usefulness of VAMS as a method for home-care sampling. These results of our proof-of-concept study should be confirmed in large series.

If our data will be confirmed, the application of this novel TDM strategy for patients on CBD may eventually result in improved quality of life for children and caregivers.

## 4. Materials and Methods

### 4.1. Chemicals and Reagents

Δ9-THC 1.0 mg/mL in methanol solution (T-005 Cerilliant), deuterated THC (9-THC-D3) 100 μg/L in methanol solution (T-003 Cerilliant; used as THC internal standard [IS]), CBD 1.0 mg/mL in methanol solution (C-045 Cerilliant), deuterated CBD (CBD-D3) 100 μg/mL in methanol solution (C-084 Cerilliant; used as CBD IS), THC acid (THC-A) 1.0 mg/mL in acetonitrile (T-093 Cerilliant) and CBD acid (CBD-A) 1.0 mg/mL in acetonitrile (C-144 Cerilliant) were purchased from Sigma-Aldrich Srl (Milan, Italy). All compounds have 98% purity. HPLC grade acetonitrile, methanol, and formic acid (99.9%) were purchased from Merck (Darmstadt, Germany). Water was purified by reverse osmosis and filtration through a Milli-Q purification system (Millipore, MA, USA).

### 4.2. Sample Collection

Samples were obtained from five patients (age 6–26 years; 1 female and 4 males) with a rare form of epileptic encephalopathy (Dravet syndrome) followed-up at ‘Giannina Gaslini’ Children’s Hospital and treated with the FDA/EMA-approved purified form of CBD (Epidiolex^®^) given for compassionate use.

Three different samples were obtained for each patient: venous blood, plasma, and capillary blood. Venous blood was obtained by venipuncture and collected in ethylenediaminetetraacetic acid (EDTA) tubes. An aliquot was used for the preparation of VAMS samples in triplicate and the remaining blood was centrifuged at 2000 g for 5 min at room temperature (RT) for obtaining plasma. Capillary blood was collected by using 30 µL VAMS (MITRA^®^, Neoteryx, Torrance, CA, USA) in triplicate at assumed steady-state conditions were included, based on blood samples that were drawn drug-fasting in the monitoring as a standard procedure.

Blood samples were also obtained from healthy adult donors and used for the preparation of calibrators and quality control (QC) samples. The hematocrit (HCT) value was measured for both patients and donors with a haematology analyzer (Advia 2120i haematology system, Siemens, Milan, Italy) and ranged 45–47%.

The study was approved by the Regional Ethical Committee (CER Liguria: 056/057/058/059-2019) and written informed consent was signed by patients or caregivers.

### 4.3. Preparation of VAMS Samples from Venous Blood

VAMS (MITRA^®^, Neoteryx, Torrance, CA, USA) samples were generated following the manufacturer’s instructions by using the 30 µL device model. The upper part of the tip was dipped into a volume of blood being careful not to completely immerse the tip into the blood to prevent overfilling. Upon turning completely red, the tips were held in place for an additional 2 seconds. Subsequently, the devices were positioned in a dedicated rack protected from light to prevent samples from touching each other while being air-dried for 1 h at room temperature.

### 4.4. Preparation of Calibrators and Quality Control Samples

Working solutions of standards and deuterated IS mixture were prepared by diluting each analyte in methanol. Calibrators and QC were made by spiking analytes from different batches of working solution into a pool of whole blood (WB). A 9-point calibration curve was prepared by adding THC and CBD to yield concentrations that ranged from 1 to 800 µg/L and included the LLOQ. QC samples were prepared by adding CBD and THC at the following four concentration levels (LLOQ, QC low, QC medium and QC high) to a pool of whole blood: 1, 3, 400, 650 µg/L.

### 4.5. Analyte Extraction from VAMS

VAMS tips were placed in 1.5 mL Eppendorf tubes and analytes were extracted with 200 µL methanol. An aliquot (10 µL) of 100 µg/L IS was added into the tube. After 10 min of incubation at 37 ± 1 °C the samples were then sonicated for 10 min at amplitude 10 microns by using a Soniprep 150 (MSE Ltd., London, UK) and then centrifuged for 10 min at 14.000 rpm and the supernatant was then transferred in an autosampler vial.

### 4.6. Chromatographic Separation

LC-MS/MS analysis was performed on a TSQ Quantiva™ Triple Quadrupole coupled to an Ultimate 3000 UHPLC Dual-Gradient Pumps (Thermo Fisher Scientific, Milan, Italy).

Starting from a previously published method [31], we added an online sample purification step. On pump 1 a solid phase extraction (SPE) online purification was carried out on a TurboFlow HTLC C18-XL column (0,5 x 50 mm, Thermo Fisher Scientific, Milan, Italy) with mobile phase A consisting of 100% water, mobile phase B of acetonitrile: isopropanol: acetone 1:1:1 and mobile phase C of 0.2% formic acid in H_2_O:MeOH 20:80. On pump 2 a gradient separation chromatography was carried out on a Waters Acquity HSS T3 column (150 mm × 2.1 mm, i.d. 1.8 μm, Waters SpA, Milan, Italy) with mobile phase A consisting of 0.1% formic acid in water, and mobile phase B of 0.1% formic acid in acetonitrile. Injection volume was 50 µL and total run time was 7.5 min.

**Figure 3:**
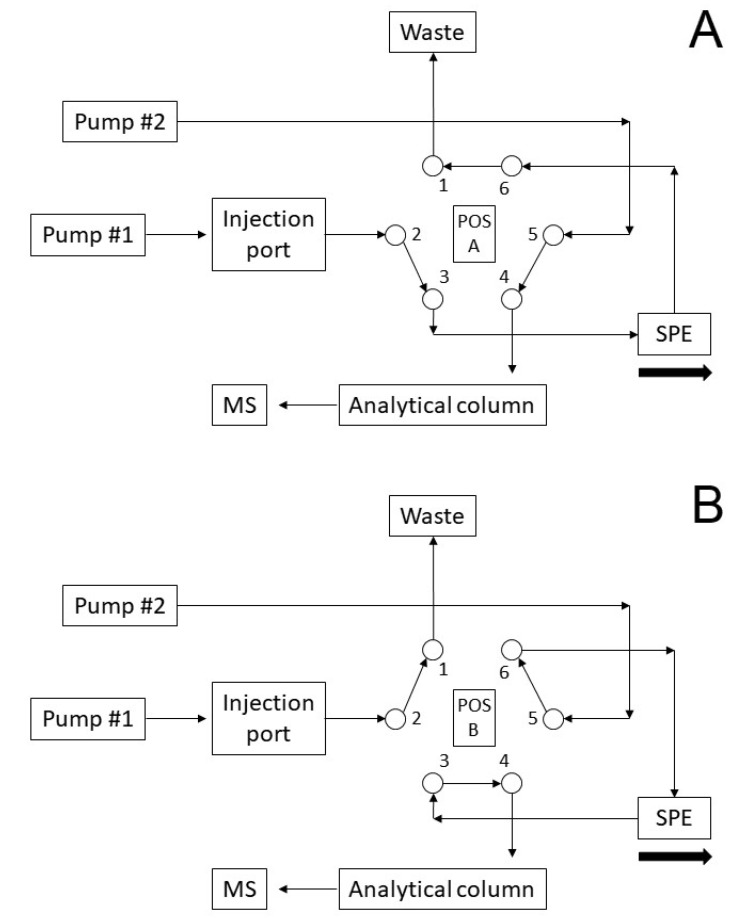
Operation scheme of the valve for online SPE.

The valve scheme is shown in Figure 3 and schematized in Table 4. Briefly, in phase 1 the valve is in position A, the sample is loaded onto the SPE column and purified. In the meantime, the chromatographic column is conditioned. In phase 2, the valve in position B allows the counter flow transfer of the analytes to the chromatographic column and the SPE column washes. In phase 3 the valve returns to position A, the chromatographic column completes its gradient and the SPE column is conditioned for the next injection. Changes in mobiles phase composition are made in rapid mode for SPE column and in slow gradient for chromatography.

### 4.7. Mass Spectrometric Detection

Mass spectrometric conditions were those previously described [31]. Briefly, ionization was achieved using atmospheric pressure chemical ionization (APCI) in the positive ion mode and analytes were detected using selective reaction monitoring (SRM) of the specific transitions, THC and CBD [M + H] + 315.2 → 193.1 *m*/*z* and their deuterated IS [M + H] + 318.2 → 196.1 *m*/*z*, respectively, with a dwell time of 249 ms.

### 4.8. Method Validation

A laboratory scheme based on the EMA guidelines was used for assay validation for both CBD and THC from VAMS samples. All the validation experiments were performed by using blood from healthy donors with HCT 45–47% which corresponded to the HCT of patients.

#### 4.8.1. Selectivity

Selectivity was evaluated by analyzing samples from six healthy volunteers not assuming drugs and on leftover samples of patients assuming antiepileptic drugs but not assuming CBD or cannabis-related products. Moreover, selectivity was also tested on samples obtained from patients under therapy with cannabis oily galenic preparations. A blank sample, a sample spiked with the two analytes at the LLOQ, and a sample spiked with IS were analyzed for each batch. Each sample was used to prepare VAMS and extracted. The absence of interfering components was accepted when the signal was less than 20% of the LLOQ for the analytes and less than 5% for the IS.

#### 4.8.2. Carry Over

The presence of carry-over was assessed by injecting blank samples in triplicate after the highest calibration standard. The signal in the blank sample following the higher standard should not be greater than 20% of the LLOQ and 5% for the IS.

#### 4.8.3. Matrix Effects and Extraction Recovery

Matrix effect was determined by comparing peak area of QC low and QC high after extraction to peak area of pure solutions at the same concentration. Recovery was determined by comparing peak area of the analytes spiked before extraction to peak area of the analytes spiked after extraction.

#### 4.8.4. Linearity

Linearity was evaluated by analyzing the calibration curve three times on three non-consecutive days. The peak area ratio of analyte/IS vs. the analyte concentration of each calibration standard were plotted by using a 1/× weighting factor. We use this weighting factor because the absolute variation is larger for higher concentrations and the data at the high end of the calibration curve tend to dominate the calculation of the linear regression. This often results in excessive error at the bottom of the curve. [41]. The mean calibration line statistics were Y = 9.62 × 10^−3^ + 9.29 × 10^−3^ × X + 9.28 × 10^−6^ × X^2^ with R^2^ = 0.9995 for CBD and Y = 3.43 × 10^−3^ + 6.28 × 10^−3^ × X + 1.95 × 10^−5^ × X^2^ with R^2^ = 0.9977 for THC. The acceptance criteria for the variation of the amounts of back-calculated standards was ± 15% of the theoretical value (except ±20% for the lowest standard).

#### 4.8.5. Precision, Accuracy and LLOQ

Within-run and between-run precision and accuracy were estimated by using the four-level QC samples and by analyzing each sample at six separate times. Between-run precision was calculated by repeating the test six times on three separate days. Accuracy was expressed as the mean relative error (expressed as a percentage) and precision as the coefficient of variation (CV%). The results were all within the acceptable ranges, i.e., ≤15% and 85–115% of the nominal concentrations for precision and accuracy respectively. The LLOQ was defined for all the analytes as the lowest concentration that could be measured with a precision ≤20% and accuracy within 80–120% of the nominal concentration. Moreover, the LLOQ should have a signal to noise ratio >5. Dilution integrity was evaluated by diluting 2 and 5-fold (*v*/*v*) the highest calibration standard with blank matrix. Each diluted sample was analyzed in triplicate.

#### 4.8.6. Stability

Stability was determined from three different assays of QC low and QC high in VAMS after maintaining them at −20 °C, +4 °C and +25 °C for 1, 2, 3 and 4 weeks. As suggested by EMA guidelines, stability was considered acceptable if the percentage difference, calculated as the ratio between the concentration measured at each sampling point and the initial concentration, was lower than 15%.

### 4.9. Statistical Methods

Comparison of results obtained from different matrices, i.e., capillary vs both venous VAMS or plasma, was made by the Mann–Whitney U test. All statistical tests were two-sided and a significance level of 0.05 was used. The software “Medcalc” (Medcalc software Ltd., Ostend, Belgium) was used for the analyses.

## 5. Conclusions

We describe the development and validation of a LC–MS/MS method for the quantification of THC and CBD from VAMS. Moreover, we show the applicability of the method on capillary blood of patients under treatment with cannabidiol. The method is suitable for TDM and personalization of therapy, especially in patients with Dravet Syndrome or Lennox-Gastaut syndrome.

## Figures and Tables

**Table 1:** Results of Intra-day and inter-day accuracy and reproducibility assays. The quality controls concentrations are respectively 1, 3, 400, 650 µg/L for LLOQ, QC low, QC medium and QC high.

	**INTER-DAY**					
	**CBD**			**THC**		
	**Dev. Std. (σ)**	**CV %**	**Accuracy %**	**Dev. Std. (σ)**	**CV %**	**Accuracy %**
**LLOQ**	0.01	6%	−13%	0.01	5%	−12%
**QC low**	0.22	10%	14%	0.32	9%	12%
**QC medium**	0.57	14%	−4%	0.52	14%	−7%
**QC high**	0.48	5%	−5%	0.50	5%	−1%
	**INTRA-DAY**					
	**CBD**			**THC**		
	**Dev. Std. (σ)**	**CV %**	**Accuracy %**	**Dev. Std. (σ)**	**CV %**	**Accuracy %**
**LLOQ**	0.01	6%	14%	0.03	14%	13%
**QC low**	0.02	11%	9%	0.08	4%	6%
**QC medium**	0.07	6%	12%	0.11	10%	11%
**QC high**	1.48	13%	6%	0.40	4%	5%

**Table 2:** Stability of CBD and THC measured on VAMS. Results are expressed as accuracy and CV percentage.

CBD				THC			
T − 20 °C		T + 25 °C		T − 20 °C		T + 25 °C	
**7 days**		**7 days**		**7 days**		**7 days**	
LLOQ	91% (6%)	LLOQ	97% (8%)	LLOQ	94% (3%)	LLOQ	97% (8%)
QC high	92% (7%)	QC high	94% (1%)	QC high	94% (1%)	QC high	94% (1%)
**14 days**		**14 days**		**14 days**		**14 days**	
LLOQ	99% (8%)	LLOQ	94% (6%)	LLOQ	91% (1%)	LLOQ	94% (6%)
QC high	97% (6%)	QC high	91% (4%)	QC high	92% (5%)	QC high	91% (3%)
**21 days**		**21 days**		**21 days**		**21 days**	
LLOQ	98% (4%)	LLOQ	98% (4%)	LLOQ	93% (5%)	LLOQ	98% (6%)
QC high	99% (3%)	QC high	90% (3%)	QC high	93% (6%)	QC high	90% (9%)
**28 days**		**28 days**		**28 days**		**28 days**	
LLOQ	99% (3%)	LLOQ	97% (6%)	LLOQ	94% (8%)	LLOQ	97% (4%)
QC high	98% (8%)	QC high	91% (8%)	QC high	98% (3%)	QC high	91% (6%)

**Table 3:** Characteristics of patients and results of CBD concentrations (expressed in µg/L) measured in plasma (p), venous (v) VAMS and capillary (c) VAMS.

	Dose (mg/kg/day)	Age (years)	Gender	CBD p (µg/L)	CBD VAMS v (µg/L)	CBD VAMS c (µg/L)
Patient 1	17.5	6	female	374	383.45	330.14
Patient 2	10.0	12	male	119	163.1	153.15
Patient 3	17.5	17	male	143	141.27	112.22
Patient 4	20.0	26	male	169	190.21	122.31
Patient 5	10.0	8	male	64	72.15	52.19

**Table 4:** Scheme of online SPE protocol.

Time (min)	Divert Valve position	Pump #1				Event SPE	Pump #2	Event Analytical Column		
		Flow rate (mL/min)	A%	B%	C%		Flow rate (mL/min)	A%	B%	
0.00	A	1.0	100	0	0	Loading	0.4	50	50	Equilibration
0.10		1.0	100	0	0					
0.10		2.5	0	100	0	Washing				
0.25		2.5	0	100	0					
0.25		1.0	100	0	0					
0.40	B					Transfer	0.4	50	50	Loading
0.90	A									
1.00							0.4	0	100	Separation
1.50		1.0	100	0	0					
1.50		2.0	0	0	100	Washing				
4.00		2.0	0	0	100					
4.00		1.0	100	0	0	Equilibration				
5.00							0.4	0	100	
5.00							0.4	50	50	Equilibration
7.50	Stop Run									
